# Trafficking of some old world primate TRIM5α proteins through the nucleus

**DOI:** 10.1186/1742-4690-8-38

**Published:** 2011-05-15

**Authors:** Felipe Diaz-Griffero, Daniel E Gallo, Thomas J Hope, Joseph Sodroski

**Affiliations:** 1 Department of Microbiology and Immunology, Albert Einstein College of Medicine, Bronx, NY 10461, USA; 2 Department of Cancer Immunology and AIDS, Dana-Farber Cancer Institute, Department of Pathology, Division of AIDS, Harvard Medical School, Boston, MA 02115, USA; 3 Department of Immunology and Infectious Diseases, Harvard School of Public Health, Boston, MA 02115, USA; 4 Department of Cell and Molecular Biology, Northwestern University, Chicago, IL 60611, USA

**Keywords:** Restriction factor, intracellular localization, retrovirus, leptomycin B

## Abstract

**Background:**

TRIM5α and TRIMCyp are cytoplasmic proteins that bind incoming retroviral capsids and mediate early blocks to viral infection. TRIM5 proteins form cytoplasmic bodies, which are highly dynamic structures. So far, TRIM5 proteins have been found only in the cytoplasm of cells. Interestingly, other proteins from the TRIM family localize to the nucleus. Therefore, we tested the possibility that TRIM5 proteins traffic to the nucleus and the impact of this trafficking on retroviral restriction.

**Results:**

Here we report that the TRIM5α proteins of two Old World primates, humans and rhesus monkeys, are transported into the nucleus and are shuttled back to the cytoplasm by a leptomycin B-sensitive mechanism. In leptomycin B-treated cells, these TRIM5α proteins formed nuclear bodies that also contained TRIM19 (PML). Deletion of the amino terminus, including the linker 1 (L1) region, resulted in TRIM5α proteins that accumulated in nuclear bodies. Leptomycin B treatment of TRIM5α-expressing target cells only minimally affected the restriction of retrovirus infection.

**Conclusions:**

We discovered the ability of human and rhesus TRIM5α to shuttle into and out of the nucleus. This novel trafficking ability of TRIM5α proteins could be important for an as-yet-unknown function of TRIM5α.

## Background

Proteins of the tripartite motif (TRIM) family contain RING, B-Box and coiled-coil domains, and thus have been referred to as RBCC proteins [1]. Members of this family have been implicated in diverse processes such as cell proliferation, differentiation, development, oncogenesis and apoptosis [1,2]. TRIM proteins often self-associate and, when overexpressed, aggregate to form nuclear or cytoplasmic bodies [1].

TRIM5α is a cytoplasmic protein that is capable of restricting retrovirus infection in a species-dependent manner [3]. Variation among TRIM5α proteins in different primates accounts for the early, post-entry blocks to infection by particular retroviruses [3-7]. For example, TRIM5α proteins of Old World monkeys block human immunodeficiency virus (HIV-1) infection [3-5,7], whereas TRIM5α proteins of New World monkeys block infection by simian immunodeficiency virus (SIV_mac_) [8]. TRIM5α from humans (TRIM5α_hu_) is not as potent in restricting HIV-1 infection as Old World monkey TRIM5α, but TRIM5α_hu _potently restricts other retroviruses, e.g., N-tropic murine leukemia virus (N-MLV) and equine infectious anemia virus (EIAV) [3,4,6-8]. Owl monkeys, a New World monkey species, are unusual in not expressing a TRIM5α protein, but instead express TRIMCyp, in which the RBCC domains of TRIM5 are fused to a cyclophilin A moiety [9,10].

Variation in splicing of the *TRIM5 *primary transcript leads to the expression of TRIM5 isoforms, designated α, γ and δ [1]. The TRIM5α isoform contains, in addition to the RING, B-box 2 and coiled-coil domains, a carboxy-terminal B30.2(SPRY) domain. The B30.2(SPRY) domain is essential for the antiretroviral activity of TRIM5α [3]. In some cases, the differences in the ability of TRIM5α proteins from various primate species to restrict particular retroviruses are determined by sequences in the B30.2(SPRY) domain [11-19]. The B30.2(SPRY) domain in TRIM5α and the cyclophilin A domain in TRIMCyp allow these restriction factors to bind specifically to particular retroviral capsids [9,20-24]. Additional sequences in the B-box 2 domain contribute to higher-order self-association of TRIM5α, which allows higher avidity for the retroviral capsid [25-27]. TRIM5α proteins aggregate on the incoming retroviral capsid [28]; and, by as-yet-uncertain mechanisms, decrease the stability of the capsid [23,27,29,30].

Some TRIM proteins localize in the nucleus of cells. One example is TRIM19 (promyelocytic leukemia (PML) protein), which is a major component of nuclear domain 10 (ND10) bodies [31-33]. TRIM19 has been shown to interfere with the replication of several DNA and RNA viruses [34-41]. Both TRIM19 and TRIM5α can inhibit herpes simplex virus replication [34,40,41], and both proteins are induced by type I interferons [18,42,43]. Thus, both cytoplasmic (e.g., TRIM5α) and nuclear (e.g., TRIM19) TRIM proteins may be involved in innate resistance to viral infection.

Here we study the intracellular localization of different TRIM5α proteins and TRIMCyp after treatment of cells with leptomycin B. Leptomycin B is a specific inhibitor of the nuclear export factor CRM1 (exportin 1), which is critical for the export of proteins carrying a nuclear export sequence [44-49]. We document that TRIM5α_hu _and TRIM5α_rh _are actively shuttling between the cytoplasm and nucleus. By contrast, TRIM5α proteins from the squirrel monkey (a New World monkey) and the cow did not accumulate in the nucleus upon leptomycin B treatment. TRIMCyp from owl monkeys also localized in the cytoplasm upon treatment with leptomycin B. We investigated the contribution of the nuclear export of TRIM5α to the antiretroviral activity of the protein.

## Results

### Leptomycin B treatment results in nuclear accumulation of some TRIM5α proteins

**Figure 1:**
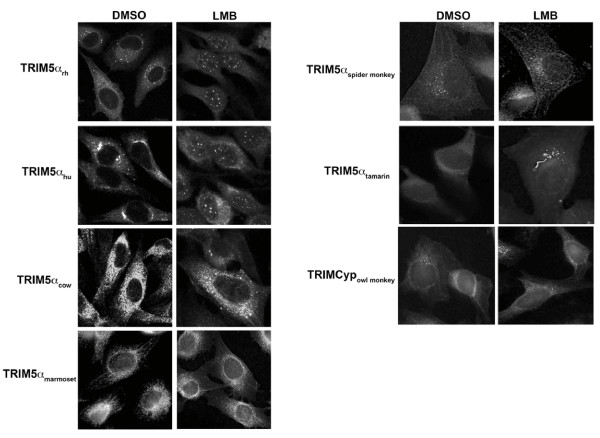
**Retention of some TRIM5 variants in the nucleus after leptomycin B treatment**. Cf2Th cells stably expressing the indicated HA-tagged TRIM5α proteins were treated with 5 ng/ml of leptomycin B (LMB) or DMSO for two hours. Treated cells were stained using anti-HA antibodies conjugated to FITC. Representative figures are shown.

During the course of studying TRIM5α, we tested the effect of leptomycin B (LMB), a specific inhibitor of nuclear export [44-49], on TRIM5α localization. As dogs do not express a functional TRIM5 protein[14], we initially studied the localization of different TRIM5α variants in canine cells. LMB treatment of Cf2Th canine cells stably expressing TRIM5α_hu _or TRIM5α_rh _resulted in the accumulation of these proteins in the nucleus (Figure 1). Both proteins were found in nuclear bodies after LMB treatment. By contrast, TRIMCyp and the TRIM5α proteins from cows and several species of New World monkeys (squirrel monkeys, spider monkeys, marmosets and tamarins) remained localized in the cytoplasm after LMB treatment. These results suggest that TRIM5α_hu _and TRIM5α_rh _shuttle into the nucleus and require active transport via the CRM1 protein to achieve cytoplasmic localization.

### Rapid accumulation of TRIM5α_hu _and TRIM5α_rh _in the nucleus after LMB treatment

**Figure 2:**
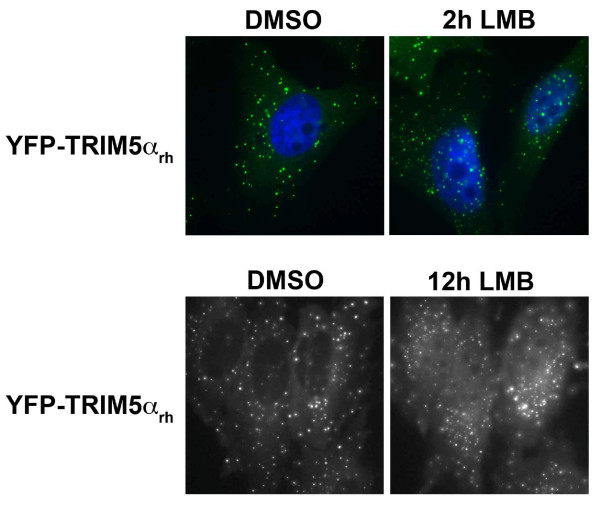
**Time course of accumulation of YFP-TRIM5α_rh _fusion protein in the nucleus after leptomycin B treatment**. HeLa cells stably expressing a YFP-TRIM5α_rh _fusion protein were treated with 5 ng/ml of LMB or DMSO for 2 and 12 hours. Treated cells were stained using anti-HA antibodies conjugated to FITC (green) and DAPI to stain the cell nucleus (blue). Representative figures are shown.

To understand the kinetics of TRIM5α_rh _movement into the nucleus, we performed time-lapse fluorescent microscopy using a HeLa cell line stably expressing a TRIM5α_rh_-yellow fluorescent protein (YFP) fusion. These experiments revealed that treatment of cells with LMB resulted in a rapid accumulation of TRIM5α_rh_-GFP in the nucleus (Figure 2). Nuclear bodies containing TRIM5α_rh_-GFP were evident by 2 hours following the initiation of LMB treatment.

### Nuclear TRIM5α_hu _and TRIM5α_rh _proteins localize to ND10 bodies with TRIM19

**Figure 3:**
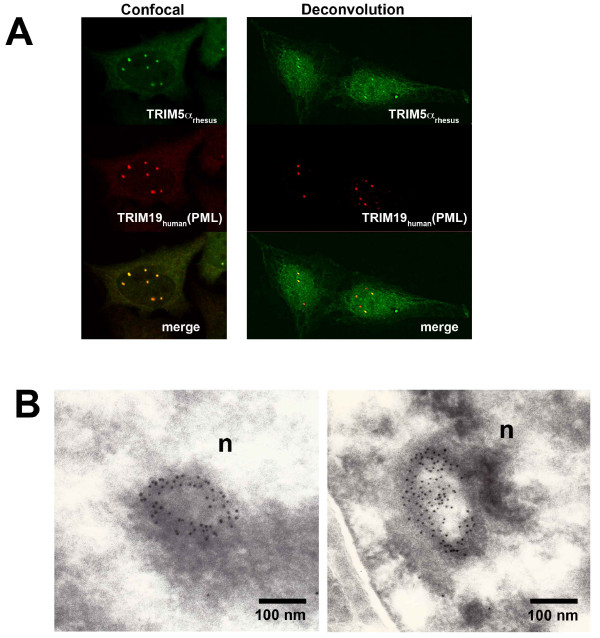
**Colocalization of TRIM5α and TRIM19 (PML) in leptomycin B-treated cells**. HeLa cells stably expressing HA-tagged TRIM5α_rh _proteins were treated with 5 ng/ml of leptomycin B (LMB) for two hours. Cells were stained for TRIM5α using anti-HA FITC-conjugated antibodies. PML was stained using anti-PML antibodies and Cy3-conjugated anti-goat secondary antibodies (A). LMB-treated HeLa cells expressing TRIM5α_rh _were fixed. Ultrathin sections were labeled using an anti-HA antibody and Protein A-gold (10-nm particles). Ring-like structures (n, nuclear bodies) in the cell nucleus were labeled with the antibody (B).

To examine whether TRIM5α_rh _localizes to the same ND10 bodies as TRIM19 after LMB treatment, LMB-treated human cells stably expressing TRIM5α_rh _were stained with antibodies directed against TRIM19 and the hemagglutinin (HA) epitope tag on TRIM5α_rh_. The nuclear TRIM5α_rh _colocalized with TRIM19 (Figure 3A). Gold-labeled antibodies directed against the HA epitope tag on TRIM5α_rh _were used to investigate the structure of the nuclear bodies. The TRIM5α-directed antibodies formed ring-like structures similar in appearance to those previously described for TRIM19 in ND10 bodies (Figure 3B) [31,33].

### Localization of a TRIM5α_rh_-pyruvate kinase fusion protein

**Figure 4:**
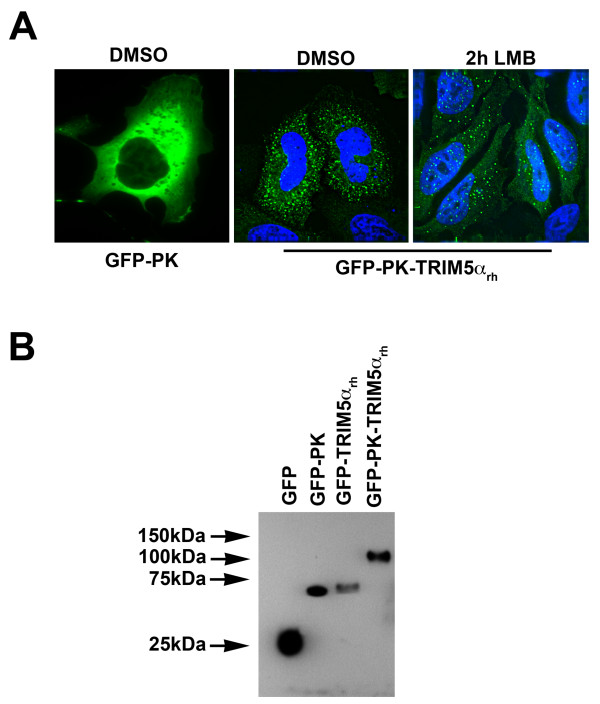
**Localization of a GFP-PK-TRIM5α protein in leptomycin B-treated cells**. HeLa cells transiently expressing the fusion constructs GFP-PK or GFP-PK-TRIM5α_rh _were treated with 5 ng/ml of LMB or with the equivalent concentration of DMSO for 2 hours (A). Protein expression levels of the different fusion constructs were measured by Western blot using anti-GFP antibodies (B).

The diameter of the nuclear pore is approximately 0.9 nm, which allows globular proteins less than 60 kD to diffuse freely through the channel [50-52]. TRIM5α proteins (approximately 55 kD) are close to this diffusion limit. Moreover, TRIM5α forms a stable dimer [20,21]; however, we do not know if the majority of TRIM5α molecules that enter the nucleus are monomers or dimers. In addition, the molecular shape of TRIM5α is unknown. These uncertainties raised the possibility that TRIM5α is actively transported into the nucleus. To test this possibility, TRIM5α_rh _was fused to pyruvate kinase (PK), which is normally a cytoplasmic protein [53] and to the green fluorescent protein (GFP) to create the GFP-PK-TRIM5α_rh _chimeric protein. The GFP-PK-TRIM5α_rh _protein and a control GFP-PK protein were transiently expressed in HeLa cells (Figure 4). Localization of these proteins was examined in untreated and LMB-treated cells (Figure 4). After a two-hour treatment with 10 nM LMB, the GFP-PK-TRIM5α_rh _protein was detected in both the nucleus and the cytoplasm. By contrast, the GFP-PK protein was detected only in the cytoplasm of untreated and LMB-treated cells. These results are consistent with the active transport of TRIM5α_rh _to the nucleus.

### Identification of TRIM5α_rh _regions modulating localization

**Figure 5:**
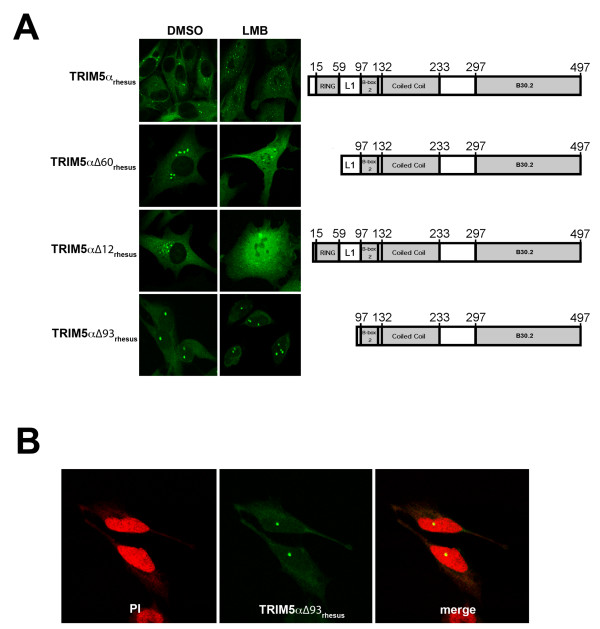
**Localization of TRIM5α_rh _N-terminal deletion mutants in leptomycin B-treated cells**. Cf2Th cells stably expressing wild-type TRIM5α_rh _or the indicated deletion mutant were treated with 5 ng/ml of LMB or DMSO for two hours. Treated cells were stained using anti-HA antibodies conjugated to FITC. TRIM5α_rh _domains are depicted for each variant, and the numbers of the amino acid residues at the boundaries of the different domains are shown (A). L1 represents the Linker 1 region. The TRIM5α_rh_∆93 protein bodies are located in the cellular nucleus (B). Cf2Th cells expressing TRIM5α_rh_∆93 were stained using using anti-HA antibodies conjugated to FITC (green) and propidium iodide for nuclear staining (red). A representative image is shown.

Proteins that localize to the nucleus and shuttle to the cytoplasm often contain nuclear localization and nuclear export signals, respectively [44-48]. TRIM5α_hu _and TRIM5α_rh _lack an obvious nuclear localization signal [54,55], nor do they contain sequences motifs predicted to function as nuclear export signals [56]. To gain some insight into the TRIM5α_rh _sequences that modulate nuclear localization and export, a series of TRIM5α_rh _mutants with deletions in N-terminal components were studied. The TRIM5α_rh _Δ12 and TRIM5α Δ60 proteins behaved like wild-type TRIM5α_rh _with respect to localization in untreated cells (Figure 5A and Table 1). However, in the LMB-treated cells, TRIM5α_rh _Δ12 and TRIM5α Δ60 exhibited a bright, more diffuse pattern with fewer nuclear bodies when compared with wild-type TRIM5α_rh _(Figure 5A and Table 1). These results indicate that neither the immediate TRIM5α_rh _N-terminus nor the RING domain significantly influence nuclear localization and export. By contrast, the TRIM5α_rh _Δ93 mutant localized to nuclear bodies and to the cytosol, even in the absence of LMB treatment (Figure 5B and Table 1). This localization pattern did not change significantly upon LMB treatment. Thus, deletion of TRIM5α_rh _sequences between residues 60 and 93, in the Linker 1 (L1) region of the protein, appears to decrease the efficiency of nuclear export of TRIM5α_rh_.

**Table 1:** Number of TRIM5α cytoplasmic and nuclear bodies in LMB-treated cells

	Number of cytoplasmic and nuclear bodies per 100 cells					
	
	DMSO			LMB		
	
	Cytoplasmic	Nuclear	Total	Cytoplasmic	Nuclear	Total
TRIM5α_rh_	448	2	450	7	543	550

TRIM5α_rh_∆12	127	3	130*	5	128	133*

TRIM5α_rh_∆60	202	8	210*	2	78	80*

TRIM5α_rh_∆93	4	151	155	12	158	170

*Cytoplasmic and nuclear bodies of TRIM5α_rh_∆12 and ∆60 were on average larger than bodies observed for wt TRIM5α_rh _and TRIM5α_rh_∆93 proteins.

### Contribution of nuclear export of TRIM5α_hu _and TRIM5α_rh _to retroviral restriction

**Figure 6:**
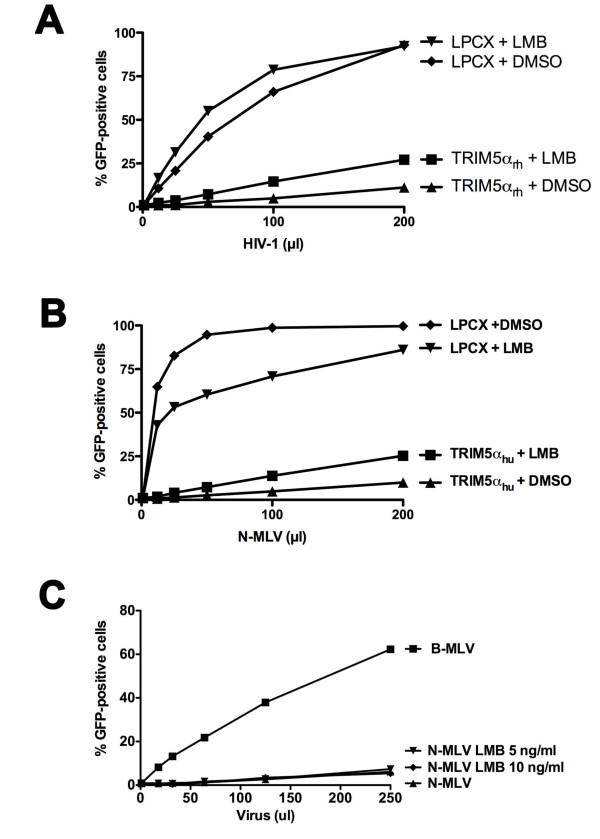
**Effect of leptomycin B treatment of TRIM5α-expressing cells on retrovirus restriction**. Cf2Th cells stably expressing TRIM5α_rh _or transduced with the empty vector LPCX were challenged with increasing amounts of HIV-1-GFP in the presence of 5 ng/ml of LMB or DMSO (A). Similarly, Cf2Th cells stably expressing TRIM5α_hu _were challenged with increasing amounts of N-MLV-GFP in the presence of 5 ng/ml of LMB or DMSO (B). TE671 cells, which naturally express TRIM5α_hu_, were challenged with B-MLV-GFP and N-MLV-GFP in the presence of the indicated concentration of LMB or the DMSO control (C). The x-axis indicates the volume of a stock of recombinant GFP-expressing virus added to the target cells. Forty-eight hours after infection, the percentage of infected cells was measured by counting the GFP-positive cells using a flow cytometer. Similar results were obtained in three independent experiments.

To study the contribution of TRIM5α nuclear export to retroviral restriction, we treated cells stably expressing TRIM5α_rh _and TRIM5α_hu _with LMB for two hours. Then the cells were challenged with recombinant HIV-1 and N-MLV expressing GFP. Treatment with LMB continued during the incubation of the cells with virus and overnight thereafter. LMB treatment exerted only minimal effects on the ability of TRIM5α_rh _to restrict HIV-1 infection and on the ability of TRIM5α_hu _to inhibit N-MLV infection (Figure 6).

## Discussion

All characterized TRIM5α proteins are located in the cytoplasm of expressing cells [15,28,57-59]. Here we report the surprising observation that some TRIM5α proteins are imported into the nucleus and then exported back into the cytoplasm by a CRM1-dependent mechanism. Of interest, this transient routing through the nucleus was observed for the TRIM5α proteins of two Old World primates, and not for the TRIM5α proteins of a cow or several New World monkeys, or for the TRIMCyp protein of another New World monkey (the owl monkey). This raises the possibility that nuclear shuttling represents a property that was gained by Old World primate TRIM5α proteins after the divergence from the New World monkeys.

Our results with the GFP-PK-TRIM5α_rh _fusion protein suggest that TRIM5α_rh _is actively transported into the nucleus, as the fusion protein is well above the size limit for passive diffusion of proteins through the nuclear pore [50-52]. Nonetheless, no typical nuclear localization motif is evident on TRIM5α [54,55]. The accumulation of TRIM5α_hu _and TRIM5α_rh _in the nucleus after LMB treatment implicates a CRM1-dependent process in the export of these TRIM5α proteins from the nucleus [44-49]. However, there are no classical nuclear export motifs in TRIM5α proteins [56]. It is possible that TRIM5α utilizes unusual motifs for interacting with nuclear pore proteins. Analysis of the localization of N-terminally truncated TRIM5α_rh _mutants suggests that deletion of residues 60-93, in the linker 1 (L1) region, disrupts the nuclear export of the protein. Whether this is a result of deletion of a non-canonical nuclear export signal or an indirect effect requires further investigation. As an example of the latter effect, the linker 1 (L1) regions could mediate the association of TRIM5α_rh _and TRIM5α_hu _with another factor that shuttles between the nuclear and cytoplasm.

Despite the accumulation of TRIM5α_hu _and TRIM5α_rh _proteins in the nucleus after LMB treatment, restriction of N-MLV and HIV-1, respectively, remained potent. Although it is possible that nuclear TRIM5α_hu _and TRIM5α_rh _can inhibit retrovirus infection, the specific recognition of the retroviral capsid, which does not enter the intact nucleus, is thought to be important for potent restriction [22,23]. A more likely explanation is that the residual TRIM5α protein in the cytoplasm of these overexpressing cells is sufficient to inhibit virus infection. Any newly synthesized TRIM5α in these cells that has not yet entered the nucleus is potentially available for capsid interaction.

One caveat of these studies is the use of exogenously expressed TRIM5α proteins to study nuclear shuttling. When better antibodies against endogenous TRIM5α become available, the shuttling behavior of the endogenously expressed TRIM5α protein can be examined.

What might be the possible advantage of having the Old World primate TRIM5α proteins shuttle into and out of the nucleus? If these TRIM5α proteins acquire post-translational modifications or binding partners in the process, our results suggest that such acquisition is apparently not necessary for HIV-1 or N-MLV restriction. The presence of TRIM5α in the nucleus could be important for other TRIM5α functions besides retroviral restriction. For example, Old World monkey TRIM5α proteins have recently been shown to inhibit the infection of herpes simplex viruses 1 and 2 [41]. The colocalization of nuclear TRIM5α in ND10 bodies with TRIM19, which also has anti-herpes virus activity [34,39,40], might have functional importance in this respect. Future studies should shed light on these interesting possibilities.

## Conclusions

Here we discovered the ability of human and rhesus TRIM5α to shuttle into and out of the nucleus. Although not essential for retroviral restriction, this novel ability of TRIM5α might be involved in other functions such as the ability of TRIM5 to trigger NF-kB[38].

## Methods

### Plasmid construction

The plasmids used to establish cell lines stably expressing TRIM5α variants or TRIMCyp have been previously described [8,58]. The plasmids expressing mutant TRIM5α_rh _proteins with N-terminal deletions were constructed by polymerase chain reaction (PCR) amplification of *TRIM5 *cDNA, as previously described [3]. The amplified fragments were cloned into the EcoRI and Cla I sites of the pLPCX plasmid (Stratagene). All of the TRIM5α proteins have an epitope tag from influenza hemagglutinin (HA). Human TRIM5α has the HA tag at the carboxyl terminus, and all the other TRIM5α proteins have the HA tag at the amino terminus.

### Creation of cells stably expressing TRIM5α and TRIMCyp variants

Retroviral vectors encoding TRIM5α or TRIMCyp proteins were created using the pLPCX vector plasmid [3]. Recombinant viruses were produced in 293T cells by cotransfecting the pLPCX plasmids with the pVPack-GP and pVPack-VSV-G packaging plasmids (Stratagene). The pVPack-VSV-G plasmid encodes the vesicular stomatitis virus (VSV) G envelope glycoprotein, which allows efficient entry into a wide range of vertebrate cells.

### Protein analysis

Cellular proteins were extracted with radioimmunoprecipitation assay (RIPA) buffer (10 mM Tris, pH 7.4; 100 mM NaCl; 1% sodium deoxycholate; 0.1% sodium dodecyl sulfate [SDS]; 1% NP-40; 2 mg of aprotinin/ml; 2 mg of leupeptin/ml; 1 mg of pepstatin A/ml; 100 mg of phenylmethylsulfonyl fluoride/ml). The cell lysates were analyzed by SDS-PAGE (10% acrylamide), followed by blotting onto nitrocellulose membranes (Amersham Pharmacia Biotech). Detection of protein by Western blotting utilized monoclonal antibodies that are specifically reactive with the HA epitope tag (Roche). Detection of proteins was performed by enhanced chemiluminescence (NEN Life Sciences Products).

### Infection with recombinant viruses expressing green fluorescent protein (GFP)

Recombinant HIV-1 or N-MLV expressing GFP were prepared as described [3]. HIV-1 viral stocks were quantified by measuring reverse transcriptase (RT) activity. For infections, 3 × 10^4 ^HeLa human epithelial cells or Cf2Th canine cells seeded in 24-well plates were incubated in the presence of virus for 24 hours. Cells were washed and returned to culture for 48 hours, and then subjected to FACS analysis with a FACScan (Becton Dickinson).

### Intracellular location of TRIM5 variants

Localization of TRIM5 variants was studied as previously described [60]. Briefly, cells were grown overnight on 12-mm-diameter coverslips and fixed in 3.9% paraformaldehyde (Sigma) in phosphate-buffered saline (PBS; Cellgro) for 30 minutes. In some experiments, cells were incubated with 5 ng/ml leptomycin B (LMB) in medium for 2-10 hours prior to fixation. Cells were washed in PBS, incubated in 0.1 M glycine (Sigma) for 10 minutes, washed in PBS, and permeabilized with 0.05% saponin (Sigma) for 30 minutes. Samples were blocked with 10% donkey serum (Dako, Carpinteria, CA) for 30 minutes, and incubated for 1 hour with antibodies. HA-tagged proteins were stained using an anti-HA FITC-conjugated antibody, clone 3F10 (Roche). The TRIM19 (PML) protein was stained with an antibody against PML, sc-9863 (Santa Cruz Biotechnology, CA) and anti-goat Cy3-conjugated antibodies(Jackson ImmunoResearch, PA). Subsequently, samples were mounted for fluorescence microscopy by using the ProLong Antifade Kit (Molecular Probes, Eugene, OR). Images were obtained with a BioRad Radiance 2000 laser scanning confocal microscope with Nikon 60X N.A.1.4 optics.

### Detection of TRIM5α by electron microscopy

HeLa cells stably expressing HA-tagged TRIM5α_rh _treated with 5 ng/ml LMB for 2 h were removed from the tissue culture dish with 5 mM EDTA in PBS, pelleted, and resuspended in a small volume of 4% paraformaldehyde in 0.2 M sodium phosphate buffer, pH 7.4. Ultrathin sections were cut at -120˚C with a cryo-diamond knife. Sections were picked up from the knife with a loop dipped in a 1:1 mixture of 2.3 M sucrose and 2% methylcellulose and transferred to a carbon-coated copper grid. Grids were left floating on PBS with the section facing down. Grids were washed in PBS and blocked in 1% bovine serum albumin (BSA) in PBS for 15 min. Grids were then incubated with the anti-HA 3F10 antibody (Roche) in 1% BSA in PBS for 30 min and washed four times for 15 min in PBS. Then, the grids were incubated with Protein A-gold 10-nm particles (Jackson Immunoresearch) in 1% BSA in PBS for 20 min and washed four times for 15 min in PBS. Images were acquired using a transmission electron microscope JEOL 1200EX-80kV.

## Competing interests

The authors declare that they have no competing interests.

## Authors' contributions

FDG designed and performed experiments, wrote the manuscript. DEG designed and performed experiments. TJH designed and performed experiments. JS designed experiments and wrote the manuscript. All authors read and approved the final manuscript.
